# NADPH Oxidases Play a Role in Pathogenicity *via* the Regulation of F-Actin Organization in *Colletotrichum gloeosporioides*

**DOI:** 10.3389/fcimb.2022.845133

**Published:** 2022-06-15

**Authors:** Na Liu, Wenfeng Wang, Chaozu He, Hongli Luo, Bang An, Qiannan Wang

**Affiliations:** ^1^ Hainan Key Laboratory for Sustainable Utilization of Tropical Bioresource, College of Tropical Crops, Hainan University, Haikou, China; ^2^ Sanya Nanfan Research Institute of Hainan University, Hainan Yazhou Bay Seed Laboratory, Sanya, China

**Keywords:** actin cytoskeleton, appressorium, *Colletotrichum gloeosporioides*, NADPH oxidases, polarized growth

## Abstract

Multiunit-flavoenzyme NADPH oxidases (NOXs) play multiple roles in living cells *via* regulating signaling pathways. In several phytopathogenic fungi, NOXs are required for the polarized growth of hyphal tips and pathogenicity to host plants, but the possible mechanisms are still elusive. In our previous study, CgNOXA, CgNOXB, and CgNOXR were identified as components of the NOX complex in *Colletotrichum gloeosporioides*. The growth and the inoculation assays revealed that CgNOXA/B and CgNOXR regulate vegetative growth and are required for the full pathogenicity of *C. gloeosporioides* to *Hevea* leaves. We further demonstrated that the vital roles of CgNOXB and CgNOXR in appressorium formation and the development of invasion hyphae account for their functions in pathogenicity. Moreover, CgNOXB and CgNOXR regulate the production and distribution of ROS in hyphal tips and appressoria, control the specialized remodeling of F-actin in hyphal tips and appressoria, and are involved in fungal cell wall biosynthesis. Taken together, our findings highlight the role of NOXs in fungal pathogenicity through the organization of the actin cytoskeleton.

## Introduction

NADPH oxidases (NOXs) are membrane-associated, multiunit flavoenzymes widely present in eukaryotes. The enzymes catalyze the reduction of molecular oxygen to superoxide anion (O_2_•-) by transferring electrons across biological membranes using NADPH as an electron donor (Lambeth, 2004; Sumimoto, 2008). NOXs were firstly identified and known as the source of the phagocyte respiratory burst; however, in the past decades, NOXs and the reactive oxygen species (ROS) they produced have been involved in many signaling pathways (Brown and Griendling, 2009; Suzuki et al., 2011). In animal cells, a series of members of the NOX family and several regulatory subunits have been identified; these proteins are implicated in cell proliferation, cell signaling, and apoptosis (Lambeth, 2004; Sumimoto, 2008; Brown and Griendling, 2009). Plant NOXs, also known as respiratory burst oxidase homologs (RBOHs), belong to a small multigenic family (Marino et al., 2012); these enzymes play multiple roles in environmental stress response (Torres et al., 2002), plant immunity (Kadota et al., 2015), programmed cell death (Suzuki et al., 2011), and polarized growth of root hairs (Foreman et al., 2003). In filamentous fungi, several NOX isoforms and regulatory components have been identified (Heller and Tudzynski, 2011; Takemoto et al., 2011; Siegmund et al., 2015). Fungal NOXs are necessary for hyphal growth, sexual reproduction, developmental processes such as the formation of appressoria, and virulence (Egan et al., 2007; Takemoto et al., 2007; Cano-Domínguez et al., 2008; Nordzieke et al., 2019). During these developmental processes, the NOX-derived ROS plays a vital role in regulating transitions from non-polarized to polarized cell growth (Egan et al., 2007; An et al., 2016). However, the underlying signaling pathway by which NOXs regulate the polarized growth remains unclear.

The cytoskeleton is a highly organized and dynamic network that exists in all eukaryotic cells; it is composed of microfilaments, microtubules, and intermediate filaments. Of the three cytoskeletal subclasses, microfilaments, made up of linear actin polymers called F-actin, are the most dynamic. The monomeric globular actin (G-actin) could assemble into filaments, and two parallel filaments form a double helix, known as F-actin. This polymerized F-actin usually assembles to elongate at one end called barbed ends and disassemble to shorten at the opposite end called pointed ends. In fungi, three higher-order F-actin structures were firstly found in *S. cerevisiae*: patches, cables, and rings (Adams and Pringle, 1984; Kilmartin and Adams, 1984). Lately, these three F-actin structures were also identified in the filamentous fungus *Aspergillus nidulans* (Araujo-Bazán et al., 2008; Berepiki et al., 2011). Actin patches are mainly accumulated in Spitzenkörper (Spk), which is located at subapical regions of hyphal tips (Riquelme and Sánchez-León, 2014); the localization of actin patches indicates their functions in endocytosis and exocytosis and involvement in hyphal tip growth (Shaw et al., 2011; Takeshita et al., 2014). Actin cables are bundles of F-actin crosslinked by tropomyosin and fimbrin (Evangelista et al., 2002); they serve as tracks for the organelle transport and secretory vesicles (Taheri-Talesh et al., 2008; Berepiki et al., 2011; Taheri-Talesh et al., 2012), whereas actin rings participate in septum formation and are required for pathogenesis in *Magnaporthe oryzae* (Ryder et al., 2013; González-Rodríguez et al., 2016; Dulal et al., 2021).

*Colletotrichum gloeosporioides* is a notorious phytopathogenic fungus that infects over 470 plant species and causes anthracnose diseases in both aerial plant parts and the postharvest fruits (Dean et al., 2012). In our previous work, two NOX components CgNOXA and CgNOXB and a regulatory protein CgNOXR were identified in *C. gloeosporioides*. The pathogenicity assay showed that knockout of either *CgNOXB* or *CgNOXR* significantly impaired the pathogenicity of *C. gloeosporioides* (Guo and An, 2018). In this study, we set out to investigate the possible mechanism of NOX in the regulation of pathogenicity, and the results showed that CgNOXB and CgNOXR are required for the polarization of actin organization in the hyphal tip, cell wall component deposition, and appressorium formation. These findings highlight the role of NOX in pathogenicity through the organization of the F-actin network.

## Material and Method

### Fungal Strains and Culture Conditions

*Colletotrichum gloeosporioides* from *Hevea* was isolated and kept previously (BioSample: SAMN17266943). Knockout mutants Δ*CgnoxA*, Δ*CgnoxB*, and Δ*CgnoxR* were constructed in our previous study (Guo and An, 2018). The strains were kept on potato agar (PDA) or cultured in a liquid medium. For the microscope analysis, conidia were cultured on Yeast Casein Sucrose (YCS) medium (1 g l^-1^ yeast extract, 1 g l^-1^ acid-hydrolyzed casein, 2% w/v sucrose, pH 6.9).

### Construction of the Double Mutant, Complementation, and Actin-Labeled Strains

To generate a double mutant of *CgnoxA* and *CgnoxB*, the Δ*CgnoxA* strain was used as the recipient strain in which *CgnoxB* was knocked out using a split-marker strategy as described in our established protocol. Briefly, the flanking sequences of *CgnoxB* were amplified and fused with the split fragments of the neomycin phosphotransferase gene (*NPTII*) which confers resistance to Geneticin (G418) (Thermo Fisher, Waltham, MA, USA). Then the two recombinant fragments were co-transformed into protoplasts of the Δ*CgnoxA* strain for the gene knockout.

To generate the complementation strain, the vector pMD-PgTt which contains the terminator of *trpC* from *A. nidulans* and the hygromycin phosphotransferase gene (*HPT*) was used. The nucleotide sequences of *Cgnox* genes together with their native promoters were amplified and ligated into the vector pMD-PgTt, respectively. Then, the plasmids were linearized before the protoplast transformation. Positive complementation strains were named as Res-Δ*CgnoxA*, Res-Δ*CgnoxB*, and Res-Δ*CgnoxR*.

To label the actin structure, the Lifeact-EGFP-expressing strain was used as the recipient strain, and *CgnoxA*, *CgnoxB*, and *CgnoxR* were knocked out respectively as described previously (Guo and An, 2018). In addition, the double-mutant strains of *CgnoxA* and *CgnoxB* were generated as described above. Protoplast preparation and transformation were performed as described in our established protocol (Wang et al., 2018). The primers that were used are listed in **Supplementary Table S1**.

### Colony Growth Assay

For the colony growth assay, disks of mycelium with a diameter of 0.5 cm were inoculated onto the PDA medium (2 g l^-1^ NaNO_3_, 0.5 g l^-1^ KCl, 1 g l^-1^ KH_2_PO_4_, 0.5 g l^-1^ MgSO_4_·7H_2_O, 0.01 g l^-1^ FeSO_4_·7H_2_O, pH 6.9), and colony morphology and diameter were recorded. Each strain contained three replicates, and all of the experiments were performed twice.

### Pathogenicity Assay

The pathogenicity assay was carried out as described in our previous report (Gao et al., 2022). Briefly, conidia were collected, washed two times with ddH_2_O, and resuspended in a solution of 0.5% Sabouraud Maltose Broth (Difco, Franklin Lakes, NJ, USA) to a final concentration of 2 × 10^5^ conidia ml^−1^. The detached leaves from rubber tree variety 73-3-97 were used for inoculation. The leaves were divided into two groups, with one group of leaves being pre-wounded with a sterile needle and the other group without being wounded. Then, droplets (5 μl) of the conidial suspensions were inoculated onto the leaves. The leaves were kept in a moist chamber at 28°C under natural illumination for 4 days, and the disease symptoms were recorded. Each treatment contained three replicates of 10 leaves, and the entire experiment was repeated three times.

### Appressorium Formation Assay

For the calculation of the appressorium formation ratio, conidia resuspended with ddH_2_O at a concentration of 5 × 10^5^ conidia ml^-1^ were incubated on hydrophobic plastic plates. After incubation for 12 and 24 h, the germination behavior was observed using Leica DM2000 microscopy. For penetration assays, the conidium droplets (3 × 10^5^ conidia ml^-1^) were inoculated on the onion epidermis that was plated on water agar plates. After incubation for 16 h, the infection structures were observed using Leica DM2000 microscopy. Each treatment contained three replications, and the entire experiment was conducted twice.

### Quantitative RT-PCR Analysis

For the RNA extraction from vegetative mycelia, the conidial suspension was inoculated into the liquid complete medium and cultured at 120 rpm, 28°C, for 2 days. Then, the mycelium was collected, disrupted in liquid nitrogen by grinding in a mortar with a pestle, and used for RNA extraction. For the RNA extraction from appressoria, conidia suspension in ddH_2_O at a concentration of 1 × 10^6^ conidia ml^-1^ was incubated on polyester; after incubation for 24 h, appressoria were collected by an RNase-free scraper and used for RNA extraction. For RNA extraction from infectious mycelia, the conidial suspension was inoculated on rubber leaves and incubated for 3 days, then the lesion area was cut from the leaves, disrupted in liquid nitrogen, and used for RNA extraction. The RNA was extracted using the RNAprep Pure Plant Plus Kit (TIANGEN Biotech, Beijing, China). For cDNA synthesis, 1 μg of total RNA from different samples was used for reverse transcription with FastKing gDNA Dispelling RT SuperMix (TIANGEN Biotech, Beijing, China) according to the manufacturer’s recommendations. To analyze the transcription levels of *CgnoxA*, *CgnoxB*, and *CgnoxR* during different stages, a quantitative real-time PCR was performed with QuantStudio 6 (Thermo Fisher, Waltham, MA, USA) in a 20-μl reaction volume using ChamQ SYBR Color qPCR Master Mix (Vazyme, Nanjing, China). The expression levels of chitin synthase genes in the mycelium of mutant strains were analyzed as demonstrated above. The β2-tubulin-coding gene was used as an endogenous control for normalization, and relative expression levels were estimated using the 2^-ΔΔCt^ method (Livak and Schmittgen, 2001). The primers are listed in **Supplementary Table S1**.

### ROS Detection

For DAB staining, conidium droplets (3 × 10^5^ conidia ml^-1^) were inoculated on the onion epidermis that was plated on water agar plates. After incubation for 12 h, the infection structures were stained with 1 mg/ml DAB (pH3.8, 30 μl) for 12 h under darkness, then the accumulation of ROS was observed with Leica DM2000 microscopy and the average optical density (AOD) of dark-brown polymers were quantified using ImageJ software. For AOD quantification, all the images were changed to eight-bit type at first. Then we selected “Area” and “Integrity density” in “Set Measurements.” After clicking “Calibrate” in “Analyze,” we selected “Uncalibrated OD” in “Function,” and then the gray value 255 equals OD value 0, and gray value 0 becomes OD value 2.71. Each strain contained three replications, and at least 60 appressoria were measured for each replicate.

For visualization of O_2_•- production, the conidial suspension was inoculated on a YCS medium-coated glass slide and incubated in a moist chamber at 28°C of 5 h before being stained with 0.05% (w/v) NBT aqueous solution for 10 min. Then the O_2_•- production of hyphae was observed by Leica DM2000 microscopy. In order to analyze the distribution of formazan precipitate in the top of germinated hyphae of different strains, the AOD in two different zones in the hyphal tip was calculated as follows: apex and subapex 7 μm, 0–7 μm from the tip; and shank 8 μm, 7–15 μm from the tip. For each strain, more than 10 hyphae were measured in each experiment, and the entire experiment was conducted twice.

### Investigation of the Actin Filament Structure by Confocal Microscopy

To investigate the actin filament structure in germinated hypha, conidial suspensions of WT, Δ*CgnoxA*, Δ*CgnoxB*, Δ*CgnoxR*, and Δ*CgnoxAnoxB* strains expressing Lifeact-EGFP (5 × 10^5^ conidia ml^-1^) were incubated on YCS-coated glass slides for 5 h before observation under the confocal. To investigate the actin filament structure in appressoria, conidial suspensions of each strain (2 × 10^5^ conidia ml^-1^) were incubated on the hydrophobic borosilicate glass coverslips (Thermo Fisher, Waltham, MA, USA) for 24 h before observation. For diphenyleneiodonium (DPI) treatment, the DPI solution was added into conidial suspension to the concentration of 40 μmol l^-1^. For the microscope analysis, the tip of germinated hypha and appressorium were captured through the Leica TCS SP8 laser scanning confocal microscope, with excitation of 488-nm argon laser and emission wavelength range of 505–525 nm. The projection of z-stack images was performed with ImageJ (http://rsbweb.nih.gov/ij/, version 1.47g). To compare the distribution of actin filaments in the top of germinated hyphae of different strains, the fluorescence intensity in three different zones in the hyphal tip was calculated as follows: apex 2 μm, 0–2 μm from the tip; subapex 5 μm, 2–7 μm from the tip; and shank 8 μm, 7–15 μm from the tip. For each strain, more than 10 hyphae were measured in each experiment, and the entire experiment was conducted twice.

### Calcofluor White Staining

For staining with Calcofluor white (CFW), hyphae that incubated on YCS-coated glass slides as mentioned above were stained with a 10-μg ml^-1^ CFW aqueous solution (Sigma-Aldrich, Merck, USA) for 10 min in the dark. The fluorescence was imaged *via* the Leica TCS SP8 laser scanning confocal microscope, with excitation of 405-nm UV laser and emission wavelength range of 430–460 nm. The projection of z-stack images was performed with ImageJ (http://rsbweb.nih.gov/ij/, version 1.47g). Quantification of the fluorescent intensity was performed by measuring the mean gray value using ImageJ software. For each strain, more than 10 hyphae were measured in each experiment, and the entire experiment was conducted twice.

### Protoplast Release Assay

Conidial suspensions were inoculated into 100 ml liquid complete medium to the initial concentration of 10^5^ conidia ml^-1^. After incubation at 120 rpm, 28°C, for 16 h, mycelium was collected by miracloth, washed with ddH_2_O, and drained with filter paper. Then, 0.2 g mycelium was incubated in 10 ml Glucanex solution at 100 rpm, 28°C for 3 h. Then protoplasts were collected by filter with miracloth, and the concentration was measured with a hemocytometer under a microscope. Each strain contained three replications.

### Statistical Analysis

Statistical significance analyses were performed in PASW Statistics (IBM, USA). Data with a single variable were analyzed by one-way ANOVA, and mean separations were performed by Duncan’s multiple-range test. Differences at P < 0.05 were considered significant.

## Results

### CgNOXs Are Involved in Vegetative Growth and Pathogenicity

**Figure 1:**
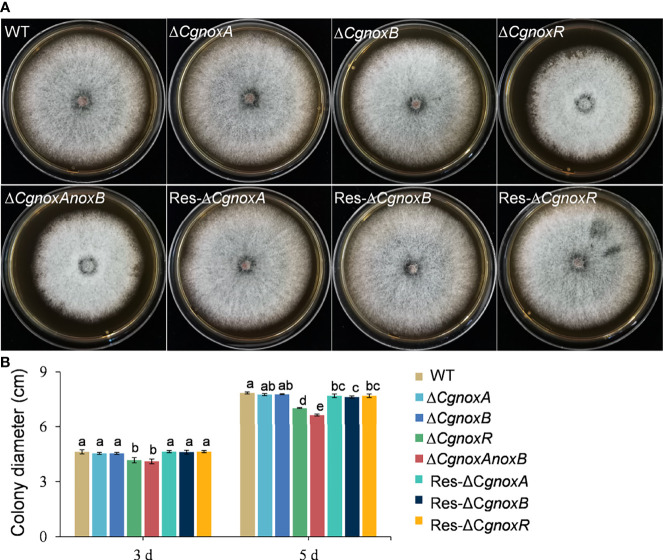
Colony morphology and diameter of *C. gloeosporioides* strains cultured on PDA medium. **(A)** Colony morphology after growth on PDA for 5 days. **(B)** Colony diameter after growth on PDA for 3 and 5 days. Bars represent standard deviations (SD).

To investigate whether CgNOXs are involved in vegetative growth, the mutant strains were cultured on PDA medium and the colony growth was recorded. After culture for 5 days, all the mutants showed a similar colony morphology to WT. The colony diameters of Δ*CgnoxA* and Δ*CgnoxB* were nearly the same as those of WT, while those of Δ*CgnoxR* and Δ*CgnoxAnoxB* were slightly decreased compared with WT (**Figure 1**). These results suggested that the CgNOX complex is required for vegetative growth.

**Figure 2:**
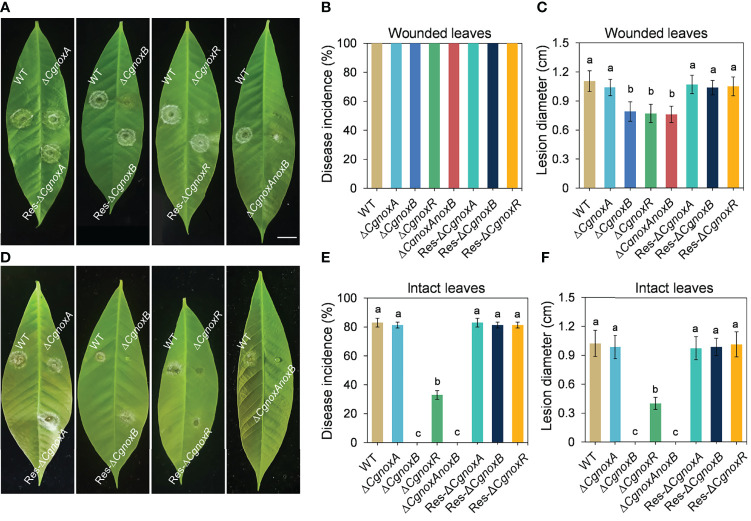
Pathogenicity assay of WT, Δ*CgnoxA*, Δ*CgnoxB*, Δ*CgnoxR*, Δ*CgnoxAnoxB*, Res-Δ*CgnoxA*, Res-Δ*CgnoxB*, and Res-Δ*CgnoxR* strains on rubber tree leaves. **(A)** Disease symptoms of pre-wounded rubber tree leaves at 4 day postinoculation (dpi). Scale bar = 1 cm. **(B)** Disease incidence of WT and mutant strains on pre-wounded leaves after 4 dpi. Values represent mean ± SD. **(C)** Mean lesion diameters on pre-wounded leaves after 4 dpi. Values represent mean ± SD. **(D)** Disease symptoms of intact rubber tree leaves at 4 day postinoculation (dpi). **(E)** Disease incidence of WT and mutant strains on intact leaves after 4 dpi. Values represent mean ± SD. **(F)** Mean lesion diameters on intact leaves after 4 dpi. Values represent mean ± SD. Columns with different letters indicate significant difference (p < 0.05).

The roles of CgNOXA, CgNOXB, and CgNOXR in the pathogenicity of *C. gloeosporioides* were investigated *via* inoculation assay on detached leaves with or without wounds. The results showed that when inoculated on the leaf wounds, all the mutants successfully infect the leaves and developed typical anthracnose lesions. However, the lesions caused by Δ*CgnoxB*, Δ*CgnoxR*, and Δ*CgnoxAnoxB* were smaller than those of WT and Δ*CgnoxA* (**Figures 2A–C**). Moreover, when inoculated on the intact leaves, the disease incidence of Δ*CgnoxR* was significantly decreased compared with that of WT and Δ*CgnoxA*, whereas Δ*CgnoxB* and Δ*CgnoxAnoxB* could not even infect the leaves at all (**Figures 2D–F**). These results revealed that CgNOXB and CgNOXR play vital roles in the pathogenicity and especially the penetration ability of *C. gloeosporioides* to host plants.

### Expression Patterns of CgNOXA, CgNOXB, and CgNOXR

**Figure 3:**
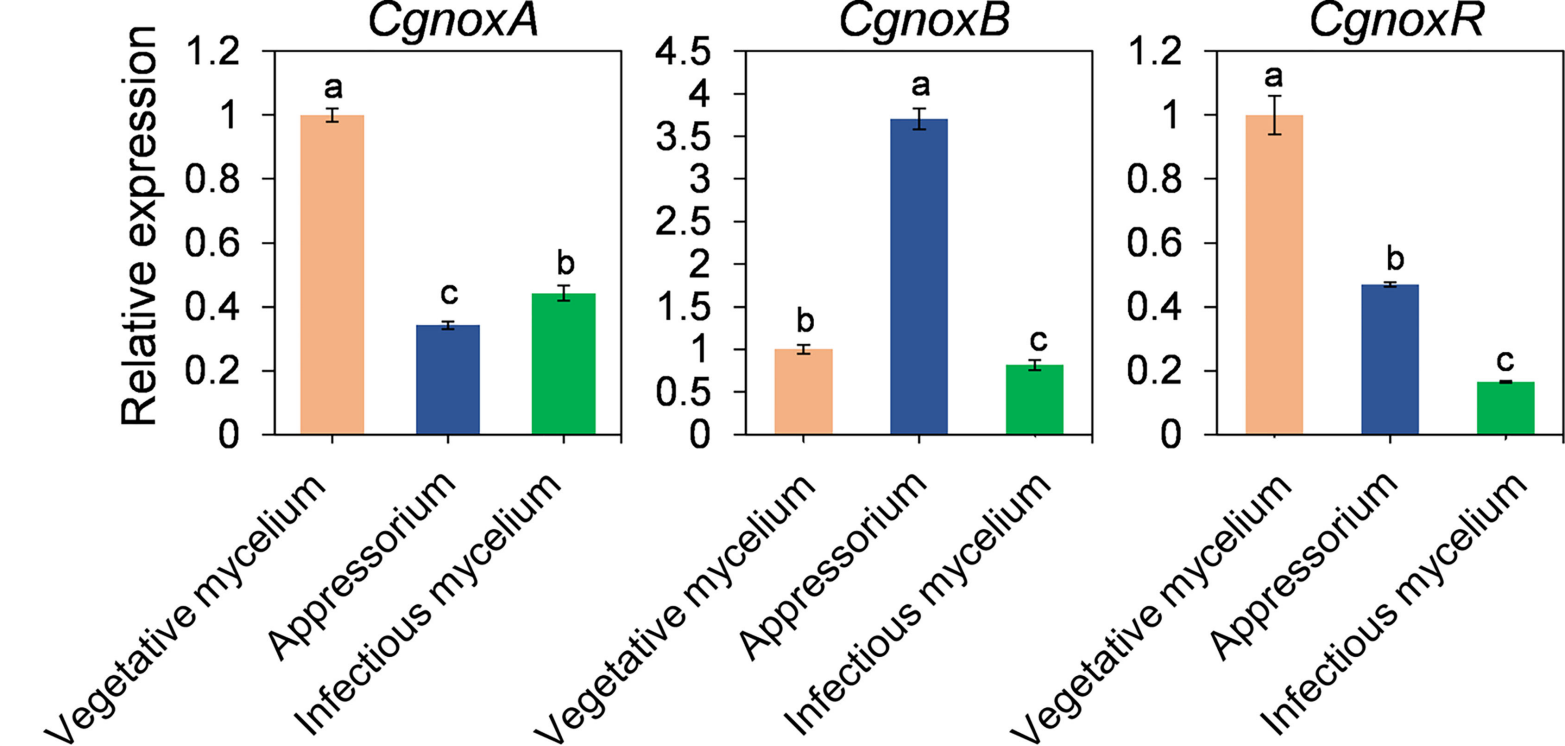
Quantitative RT-PCR of transcript of *CgNoxA*, *CgNoxB*, and *CgNoxR.* Bars represent standard deviations (SD). Columns with different letters indicate significant difference (p < 0.05).

The expression patterns of *CgnoxA*, *CgnoxB*, and *CgnoxR* during vegetative growth *in vitro*, appressorium formation, and colonization in plant leaves were investigated *via* a qRT-PCR assay. The results (**Figure 3**) revealed that in appressoria, the transcription level of *CgnoxB* was about 2.5-fold higher than that during *in vitro* growth and *in vivo* colonization, whereas the transcription levels of *CgnoxA* and *CgnoxR* were about 0.5-fold lower than in the other stages. Meanwhile, the expressions of the three genes during *in vivo* colonization were all down-regulated compared with that during *in vitro* growth. These results enlightened the role of CgNOXB in the appressorium formation of *C. gloeosporioides.*

### CgNOXB and CgNOXR Regulate Appressorium and Penetration Peg Formation

**Figure 4:**
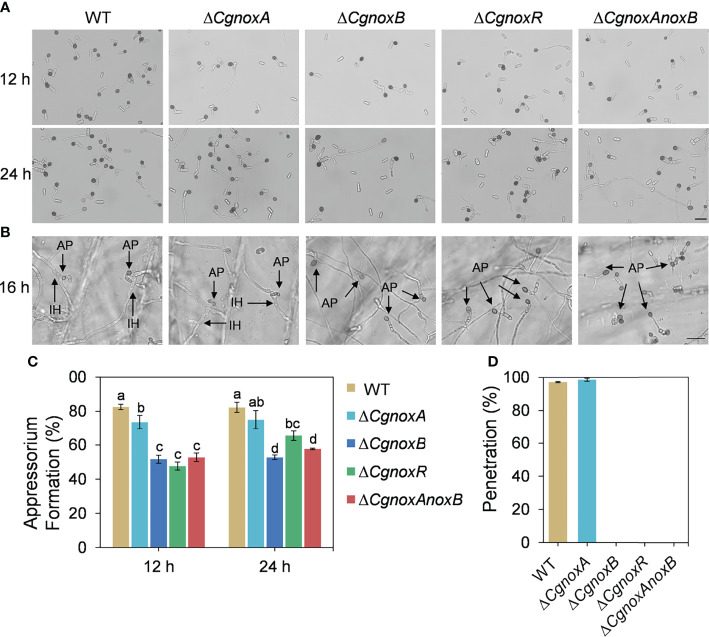
Appressorium formation on hydrophobic plastic plates and onion epidermis. **(A)** Appressorium formation assay of WT, Δ*CgnoxA*, Δ*CgnoxB*, Δ*CgnoxR*, and Δ*CgnoxAnoxB* strains incubation on hydrophobic plastic plates for 12 and 24 h. Scale bar = 20 μm. **(B)** Appressorium formation and penetration on onion epidermis after 16 h. AP: appressorium, IH: invasive hypha. Scale bar = 20 μm. **(C)** Appressorium formation rate at 12 and 24 h postinoculation. Bars represent standard deviations (SD). Columns with different letters indicate significant difference (p < 0.05). **(D)** Bar charts show the relative percentage penetration of each strain on onion epidermis at 24 hpi, assessed by recording the frequency of hyphal penetration from an appressorium (three biological replicas).

To explore the roles of NOX in appressorium formation, the germination rates and appressorium formation of the mutants were investigated. The mutants were cultured on hydrophobic plastic plates to induce appressorium formation. After incubation for 12 h, over 80% conidia of WT formed typical appressoria, whereas Δ*CgnoxA*, Δ*CgnoxB*, Δ*CgnoxR*, and Δ*CgnoxAnoxB* showed decreases in appressorium formation, with about 73.6%, 51.7%, 47.6%, and 52.8%, respectively. After incubation for 24 h, approximately 82.3% and 75.0% conidia from WT and Δ*CgnoxA* formed appressoria; in comparison, the rates of Δ*CgnoxB*, Δ*CgnoxR*, and Δ*CgnoxAnoxB* were only 52.8%, 65.5%, and 57.7%, respectively (**Figures 4A, B**). The results revealed that CgNOXs are required for appressorium development.

The formation of penetration peg was further investigated by incubation of the mutants on the onion epidermis. The result (**Figures 4C, D**) showed that, after incubation for 16 h, most of the appressoria of WT and Δ*CgnoxA* formed typical invasive hyphae (also named primary hyphae) and successfully penetrated the plant tissue. By contrast, appressoria of Δ*CgnoxB*, Δ*CgnoxR*, and Δ*CgnoxAnoxB* failed to penetrate and invade onion cells. The results suggested that CgNOXB and CgNOXR are required for the penetration ability of *C. gloeosporioides* to plant.

### CgNOXB and CgNOXR Regulate ROS Generation

**Figure 5:**
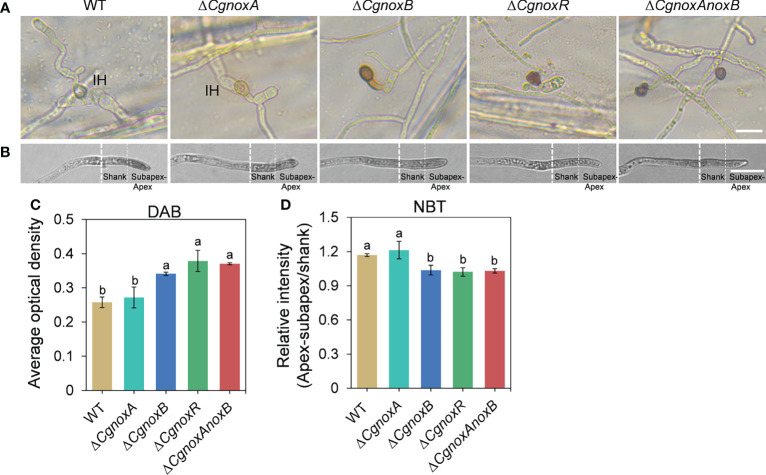
CgNOX regulates reactive oxygen species (ROS) in *C. gloeosporioides*. **(A)** DAB staining of WT, Δ*CgnoxA*, Δ*CgnoxB*, Δ*CgnoxR*, and Δ*CgnoxAnoxB* strains after inoculation on onion epidermis for 12 h. Scale bar = 10 μm. **(B)** Germinated conidia of WT, Δ*CgnoxA*, Δ*CgnoxB*, Δ*CgnoxR*, and Δ*CgnoxAnoxB* strains were stained with nitro blue tetrazolium chloride (NBT) and viewed under light microscopy. White dotted lines indicate the regions for the measurement. Scale bar = 10 μm. **(C)** The deposition of dark brown polymers in appressorium was analyzed using ImageJ software. Bar chart showing AOD (average optical density) of each strain. Bars represent standard deviations (SD). Columns with different letters indicate significant difference (p < 0.05). At least 60 appressoria from each strain were analyzed. **(D)** The AOD of formazan precipitate of apex–subapex and shank in the hyphal tips were quantified through ImageJ software. Bar chart showing the ratio of AOD of apex–subapex to shank. Bars represent standard deviations (SD). Columns with different letters indicate significant difference (p < 0.05). At least 10 hyphae from each strain were analyzed.

The appressoria of the mutants were stained with DAB to investigate the ROS generation. After incubation for 24 h on onion epidermis, WT and Δ*CgnoxA* showed a little accumulation of dark-brown polymers around appressoria. (**Figures 5A, B**), whereas for the Δ*CgnoxB*, Δ*CgnoxR*, and Δ*CgnoxAnoxB* mutants, the appressoria accumulated an amount of dark-brown polymers, with higher AOD levels than those of WT and Δ*CgnoxA* (**Figures 5A, B**). In addition, these three mutants did not form invasive hyphae as mentioned above. To further assess O_2_•-, the direct product of NOXs, the hyphae of the mutants were stained with NBT. Microscopic observation showed that the WT and Δ*CgnoxA* accumulated blue formazan precipitate intensively in the apex of hyphal tips (**Figures 5C, D**), suggesting the polarity distribution of O_2_•-, whereas for Δ*CgnoxB*, Δ*CgnoxR*, and Δ*CgnoxAnoxB*, the O_2_•- generation was uniformly distributed in the hyphal tips.

### CgNOXB and CgNOXR Regulate the Organization of F-Actin

In our previous work, a widely used Lifeact-EGFP gene fusion was introduced into *C. gloeosporioides* to observe the organization of F-actin by live-cell imaging (Liu et al., 2021). To understand the roles of CgNOXs in F-actin organization, we then generated the Δ*CgnoxA*, Δ*CgnoxB*, and Δ*CgnoxR* and Δ*CgnoxAnoxB* mutants that express Lifeact-EGFP. Then the F-actin structure in mutants was investigated.

**Figure 6:**
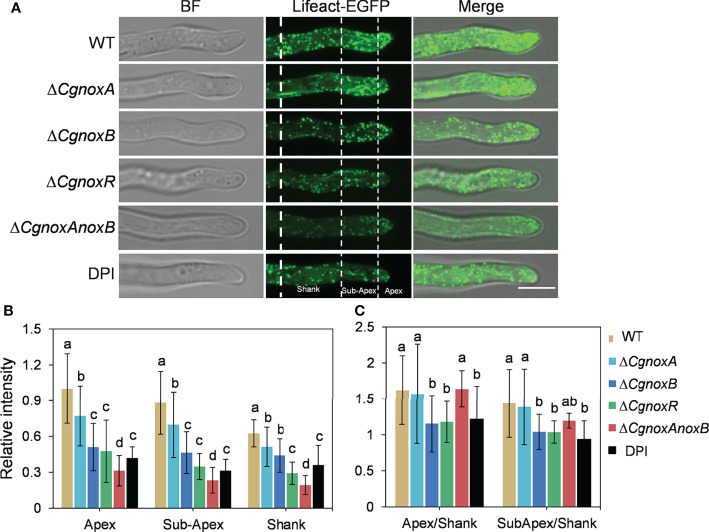
CgNOX regulates the actin filament structure in the tip of the germ tube of *C. gloeosporioides*. **(A)** Lifeact-EGFP-labeled actin filament structures in WT (DPI treatment or not), Δ*CgnoxA*, Δ*CgnoxB*, Δ*CgnoxR*, and Δ*CgnoxAnoxB* strains. The tip of germinated hypha was divided into three zones as apex (0–2 μm from tip), subapex (2–7 μm from tip), and shank (7–15 μm from tip) by white dotted lines. Scale bar = 5 μm. **(B)** The relative amount of actin filaments in the tip of germinated hypha in WT, Δ*CgnoxA*, Δ*CgnoxB*, Δ*CgnoxR*, and Δ*CgnoxAnoxB* strains expressing Lifeact-EGFP. The amount of actin filament within the apex, subapex, and shank was measured. The fluorescence intensity of the apex in WT strain was converted to 1, and the relative amount of actin filament was plotted. At least 10 hyphae were measured for each strain. Bars represent standard deviations (SD). Columns with different letters indicate significant difference (p < 0.05). **(C)** Quantification of the distribution of actin filaments in the tip of germinated hypha in WT (DPI treatment or not), Δ*CgnoxA*, Δ*CgnoxB*, Δ*CgnoxR* and Δ*CgnoxAnoxB* strains expressing Lifeact-EGFP. At least 10 hyphae were measured for each strain. Bars represent standard deviations (SD). Columns with different letters indicate significant difference (p < 0.05).

In the conidia of WT and the mutants, F-actin showed a typical filamentous network and patches, suggesting that the knockout of NOX genes did not influence the F-actin network in conidia (**Figure S1**). In hyphal tips of WT and Δ*CgnoxA*, F-actin showed a polarized distribution with patches and cable structures (**Figure 6A**), whereas in Δ*CgnoxB*, Δ*CgnoxR*, and Δ*CgnoxAnoxB* mutants, this kind of organized distribution was diminished (**Figure 6A**). To quantitate this polarity distribution of F-actin, the hyphal tips were divided into three regions of apex, subapex, and shank, and the relative fluorescence intensity was calculated. The results were in accordance with that of the microscope observation, revealing that WT and Δ*CgnoxA* employed a higher intensity at the apex and subapex regions than in the shank region, while Δ*CgnoxB*, Δ*CgnoxR*, and Δ*CgnoxAnoxB* employed uniform intensity all through the hyphal tips (**Figure 6A**). To further verify whether this kind of F-action polarity was mediated by NOXs, the WT strain was treated with DPI, the inhibitor of NOXs. The microscopic analysis showed that DPI led to a decrease in fluorescence intensity in all three zones of hyphal tips; moreover, the polarity of F-actin was also diminished by DPI.

**Figure 7:**
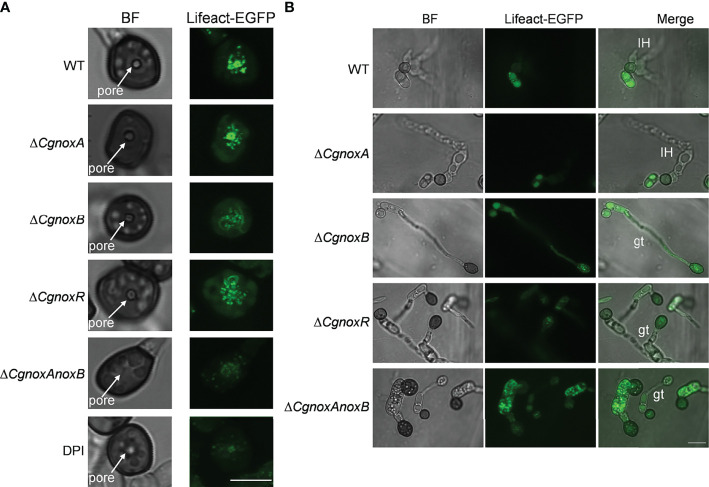
CgNOX regulates F-actin organization during appressorium development and penetration of *C. gloeosporioides*. **(A)** Micrographs of F-actin organization in appressorium visualized by expression of Lifeact-EGFP in WT (DPI treatment or not), Δ*CgnoxA*, Δ*CgnoxB*, Δ*CgnoxR*, and Δ*CgnoxAnoxB* strains. Conidial suspensions at 2 × 10^5^ conidia ml^-1^ were inoculated onto hydrophobic glass coverslips for 24 h. Scale bar = 5 μm. **(B)** Micrographs of F-actin organization in appressorium and infectious hyphae of each strain on onion epidermis. Conidial suspensions at 2 × 10^5^ conidia ml^-1^ were inoculated onto onion epidermis for 15 h. IH represents invasive hyphae, gt means germinated tube. Scale bar = 10 μm.

Then the F-actin structures in the appressoria were investigated. The results showed that in the appressoria of WT, F-actin was reorganized to a ring structure around the appressorium pore. This ring-shaped F-actin network was also observed in Δ*CgnoxA* (**Figure 7A**), whereas in Δ*CgnoxB*, Δ*CgnoxR*, and Δ*CgnoxAnoxB*, F-actin showed a diffused distribution and did not form a ring structure in appressoria. Furthermore, DPI treatment also interfered with the organization of the F-actin network, resulting in a fuzzy ring structure with very low fluorescence intensity (**Figure 7A**). During incubation on onion epidermis for 15 h, WT and Δ*CgnoxA* formed typical appressoria and invasive hyphae; furthermore, F-actin in appressoria was diffused with low intensity, suggesting that there was a reorganization of the F-actin structure in appressoria after the formation of invasive hyphae (**Figure 7B**). In comparison, appressoria of Δ*CgnoxB*, Δ*CgnoxR*, and Δ*CgnoxAnoxB* showed diffused F-actin structures of actin patches in conidia and appressoria; meanwhile, there were no invasive hyphae formed. In addition, to investigate whether the transcription of *Lifeact-GFP* was interfered in the mutants, a qRT-PCR analysis was conducted. The result showed that the relative expression levels of *Lifeact-GFP* in these mutants were all below twofold, suggesting that the knockout of NOX-coding genes did not influence the transcription of *Lifeact-GFP* (**Figure S2**). These results demonstrate the important roles of CgNOXB and CgNOXR in F-actin organization.

### CgNOXA, CgNOXB, and CgNOXR Are Required for Cell Wall Integrity

**Figure 8:**
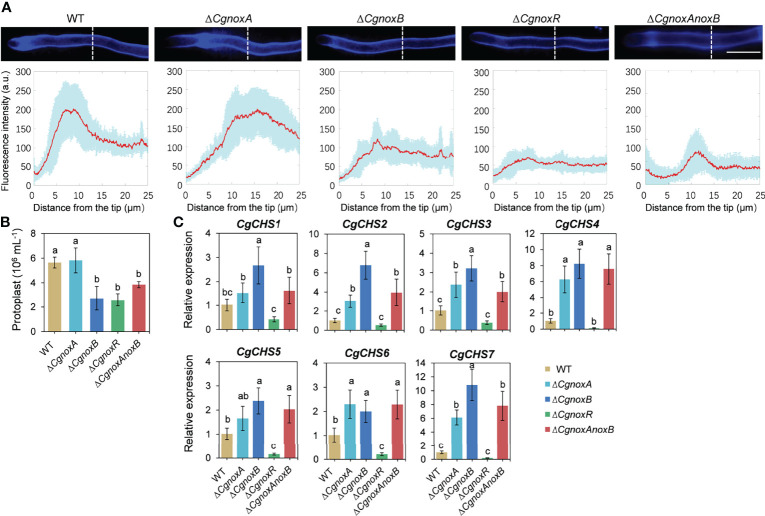
CgNOX regulates cell wall integrity of *C. gloeosporioides*. **(A)** CFW staining of hyphal tip from WT, Δ*CgnoxA*, Δ*CgnoxB*, Δ*CgnoxR*, and Δ*CgnoxAnoxB* strains. White dotted lines indicate the regions for the measurement. Scale bar = 10 μm. Quantitative measurements of the fluorescence intensity along the hypha from the tip. The red lines show the average fluorescence intensity and the blue-shaded regions indicate the SD. At least 10 hyphae were measured for each strain. **(B)** Bar chart showing frequency of protoplast generation following incubation of mycelium from each strain with lyticase. Bars represent standard deviations (SD). Columns with different letters indicate significant difference (p < 0.05). **(C)** Bar charts showing the relative expression level of seven chitin synthase genes in each strain. Bars represent standard deviations (SD). Columns with different letters indicate significant difference (p < 0.05).

To investigate the roles of CgNOXs in fungal cell wall synthesis, the hyphae of the strains were observed with Calcofluor white (CFW) staining. In WT, CFW fluorescence was intensively distributed at the apex regions of hyphal tips, indicating the polarized deposition of the cell wall material, whereas in Δ*CgnoxA*, the fluorescence is mainly located in subapex regions. Moreover, this kind of polarity was diminished in the hyphal tips of Δ*CgnoxB*, Δ*CgnoxR*, and Δ*CgnoxAnoxB*, in which the fluorescence was uniformly distributed all through the hyphal tips (**Figure 8A**).

The protoplast release assay was conducted to assess the sensitivity of the cell wall to enzymatic degradation. The results showed that Δ*CgnoxA* released a similar number of protoplasts as WT after the treatment with lyases. However, Δ*CgnoxB*, Δ*CgnoxR*, and Δ*CgnoxAnoxB* released fewer protoplasts than WT (**Figure 8B**). The results indicated the change in the cell wall composition of Δ*CgnoxB*, Δ*CgnoxR*, and Δ*CgnoxAnoxB*. Chitin synthases participate in the biosynthesis of chitin and are involved in the cell wall integrity of filamentous fungi (Yang and Zhang, 2019; Wang et al., 2021). Therefore, the expression changes of chitin synthases in the mutants were investigated. There were seven chitin synthases (*CgCHS*) identified in *C. gloeosporioides* (Wang et al., 2021). The qRT-PCR assay (**Figure 8C**) showed that in Δ*CgnoxA*, the relative expression levels of *CgCHS2*, *CgCHS3*, *CgCHS4*, *CgCHS6*, and *CgCHS7* increased over twofold; meanwhile, all seven genes were upregulated in Δ*CgnoxB*. However, in Δ*CgnoxR*, all the *CgCHS* genes were dramatically down-regulated. These data suggest that CgNOXA, CgNOXB, and CgNOXR are all involved in the cell wall integrity in *C. gloeosporioides*.

## Discussion

NOXs play important roles in many biological processes in living cells. In filamentous fungi, the NOX-derived ROS regulate many aspects of the life cycle including vegetative hyphal growth, conidiation, secondary metabolism, and pathogenicity of many phytopathogenic fungi (Egan et al., 2007; Giesbert et al., 2008; Segmüller et al., 2008; Heller and Tudzynski, 2011; Tudzynski et al., 2012; Ryder et al., 2013; Wang et al., 2020). Unlike that in *M. oryzae* and some other phytopathogenic fungi, here we found that knockout of *CgnoxA* or *CgnoxB* did not influence the colony morphology or growth rate (**Figure 1**). However, the vegetative growth of Δ*CgnoxR* and the double-mutant Δ*CgnoxAnoxB* was slightly reduced, indicating that CgNOXR plays an important role in the regulation of the vegetative growth, and CgNOXA and CgNOXB may be functionally redundant in the regulation of vegetative growth, at least partially. A pathogenicity assay was conducted subsequently. When inoculated onto the wounds of leaves, all the mutants could infect the host and cause lesions; moreover, Δ*CgnoxB*, Δ*CgnoxR*, and Δ*CgnoxAnoxB* caused smaller lesions in comparison to WT and Δ*CgnoxA*. However, when inoculated on intact leaves, Δ*CgnoxB*, Δ*CgnoxR*, and Δ*CgnoxAnoxB* lost the ability to infect the hosts (**Figure 2**). These results suggested that CgNOXB and CgNOXR play important roles in the early infection process of *C. gloeosporioides* to the host plant. As a hemibiotrophic pathogen, *C. gloeosporioides* could form the specialized infection structure appressoria to infect plant hosts. The appressoria generate high turgor and physical force to rupture plant cuticle and form a penetration peg which develops into invasive hyphae to penetrate plant tissue (De Jong et al., 1997; Ryder and Talbot, 2015). In addition, NOXs are involved in appressorium formation in several phytopathogenic fungi (Ryder and Talbot, 2015; Wang et al., 2020). The expression pattern assay of *CgnoxA*, *CgnoxB*, and *CgnoxR* showed that *CgnoxB* was significantly up-regulated in the appressoria (**Figure 3**). These results enlightened us that CgNOXs, especially CgNOXB, play important roles in appressorium formation in *C. gloeosporioides.*

Then the appressorium formation and the following infection processes of the mutants were investigated. We found that knockout of *CgnoxA* did not influence the appressorium formation and invasive hyphae development. However, knockout of *CgnoxB* or *CgnoxR* significantly inhibited the two processes (**Figure 4**). Furthermore, the development of invasive hyphae was even diminished in Δ*CgnoxB* and Δ*CgnoxAnoxB.* The result was in accordance with our hypothesis that CgNOXB plays an important role in appressorium formation in *C. gloeosporioides.* Although the transcription of *CgnoxR* was not up-regulated in appressoria, knockout of the gene did significantly interfere with the appressorium formation and invasive hyphal development. This might be because NOXR is the main regulatory component of the NOX complex (Takemoto et al., 2006; Cano-Domínguez et al., 2008). These results provided evidence for the roles of CgNOXB and CgNOXR in pathogenicity by regulation of appressorium formation and invasive hyphal development. Our results were partially consistent with findings in *M. oryzae*, in which Δ*nox1*, Δ*nox2*, Δ*noxR*, and Δ*nox1nox2* are all non-pathogenic due to their defects in the development of the penetration peg (Egan et al., 2007; Ryder et al., 2013).

In plant cells, actin organization is important for polarized growth of root tip and pollen tube growth (Gibbon et al., 1999; Miller et al., 2010). In fungi, NOXs play vital roles in the developmental processes through the regulation of transitions from non-polarized to polarized cell growth (Egan et al., 2007; Kayano et al., 2013). Moreover, the polarized fungal growth is bound up with the remodeling of the F-actin cytoskeleton (Ryder et al., 2013). Therefore, we firstly investigate the F-actin organization in germ tubes. The results showed that there is a significantly polarized distribution of F-actin in the hyphal tips (**Figure 6**), while this ordered structure was disrupted in Δ*CgnoxB*, Δ*CgnoxR*, and Δ*CgnoxAnoxB* mutants.

During the appressorium formation and maturity, the F-actin remodels the ring structure around the appressorium pore, which is vital for the development of invasive hyphae (Dagdas et al., 2012). Here we found that in WT and Δ*CgnoxA*, F-actin formed a typical ring structure around the appressorium pore. However, in Δ*CgnoxB*, Δ*CgnoxR*, and Δ*CgnoxAnoxB*, F-actin did not organize into a ring and exhibited in a diffused distribution (**Figure 7A**). Besides, we treated *C. gloeosporioides* with the NOX inhibitor DPI and found that DPI treatment led to a fuzzier ring organization of F-actin. Our results were somehow different from the findings in *M. oryzae*, in which Nox1 is necessary for the F-actin network at the appressorium pore, whereas Nox2 and NoxR are indispensable for the ring structure (Ryder et al., 2013). After the development of invasive hyphae, the appressoria of WT and Δ*CgnoxA* showed a degradation of the F-actin ring structure, whereas those of Δ*CgnoxB*, Δ*CgnoxR*, and Δ*CgnoxAnoxB* were still with strong actin fluorescence (**Figure 7B**), suggesting the detention of F-actin remodeling in the mutants.

As the oxidase complex, NOXs produce ROS to regulate downstream signaling. We then investigate the ROS generation *via* DAB and NBT staining. Visualization of O_2_•- *via* NBT showed that in WT, there is a polarized manner of O_2_•- production in hyphal tips, while knockout *CgnoxB* or *CgnoxR* weakened the polarity of O_2_•- production (**Figure 5**). Similar results were also observed in the NOX gene knockout mutants of *Aspergillus nidulans*, *Neurospora crassa*, and *Epichloë festucae* (Takemoto et al., 2006; Cano-Domínguez et al., 2008; Semighini and Harris, 2008). Besides, the DAB staining revealed that after infection into the host cells, the appressoria of WT and Δ*CgnoxA* showed less ROS production than that of Δ*CgnoxB*, Δ*CgnoxR*, and Δ*CgnoxAnoxB* (**Figure 5**). This might be because the appressoria of WT and Δ*CgnoxA* had already formed invasive hyphae and infected onion cells, making the invasive hyphae become the new “hot spot” for ROS production. In comparison, the other three mutants were detained in the appressorium stage.

F-Actin patches and cables are involved in exocytosis and vesicle secretion in fungi (Berepiki et al., 2011). Meanwhile, the hyphal growth and appressorium formation of fungi both require the biosynthesis, exocytosis, and deposition of cell wall materials (Read, 2011; Schuster et al., 2012). Since the cell wall integrity (CWI) signaling pathway plays a crucial role in fungal growth and pathogenicity (Malavazi et al., 2014), we set out to investigate whether NOX-dependent F-actin organization is involved in CWI (**Figure 8**). The CFW staining showed that in the WT strain, the CFW fluoresce was intensively located at the apex region of hyphal tips, suggesting a strong polarity in the deposition of cell wall material, whereas in *ΔCgnoxA*, the fluoresce was mainly located in the subapex region. Furthermore, in *ΔCgnoxB*, *ΔCgnoxR*, and Δ*CgnoxAnoxB*, the fluorescent signal was uniformly distributed through the hyphal tips. The result indicated that the NOX-dependent F-actin organization is important for cell wall synthesis. In addition, the protoplast release assay showed that the cell wall composition was also affected in *ΔCgnoxB*, *ΔCgnoxR*, and Δ*CgnoxAnoxB* mutants. Moreover, the following qRT-PCR analysis revealed that the relative expression levels of *CgCHS* genes were up-regulated in *ΔCgnoxA*, *ΔCgnoxB*, and Δ*CgnoxAnoxB*, which might be the main cause for cell wall composition changes in the mutants. However, for *ΔCgnoxR*, the expressions of the seven *CgCHS* genes were all significantly down-regulated, suggesting that CgNOXR might play other regulatory functions in addition to its function on the NOX complex.

**Figure 9:**
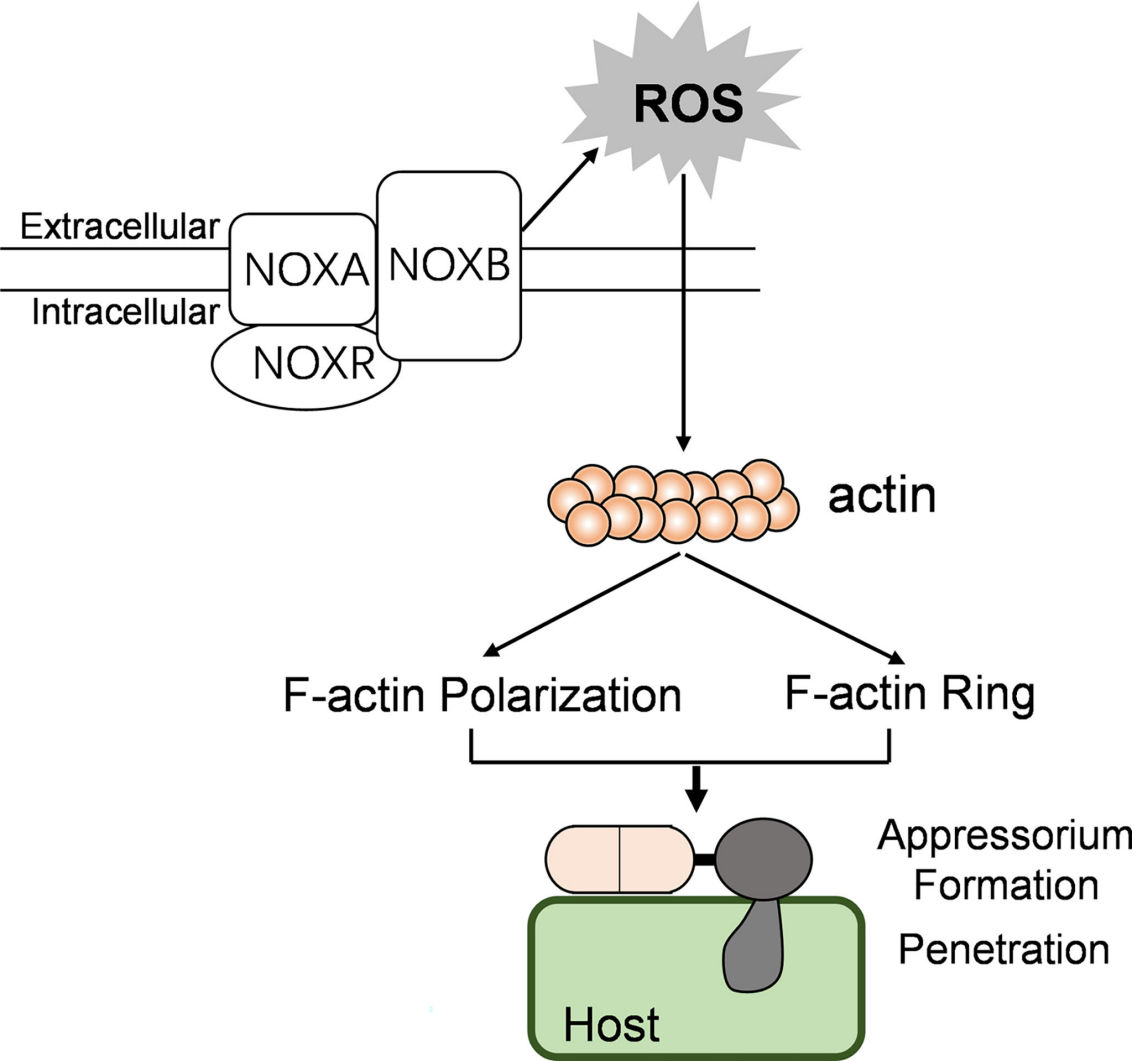
The possible model showing the relationships between NOX, F-actin organization, and pathogenicity in *C. gloeosporioides*.

Taken together, we conclude that CgNOXB and CgNOXR regulate the spatial production of ROS and are necessary for polarized F-actin organization; this polarized F-actin structure regulates cell wall integrity and controlled polarized hyphal growth, appressorium formation, and invasive hyphal development in *C. gloeosporioides* (**Figure 9**). Our study extends the understanding of the molecular mechanism by which NOXs regulate the pathogenicity of phytopathogenic fungi.

## Data Availability Statement

The original contributions presented in the study are included in the article/**Supplementary Material**. Further inquiries can be directed to the corresponding author.

## Author Contributions

QW and BA designed the study. NL, WW, and QW performed the experiments. QW, BA, and NL wrote the manuscript. CH and HL revised the manuscript. All authors contributed to the article and approved the submitted version.

## Funding

This work was supported by the National Natural Science Foundation of China (32001846, 32000102, 31860478, 32160594).

## Conflict of Interest

The authors declare that the research was conducted in the absence of any commercial or financial relationships that could be construed as a potential conflict of interest.

## Publisher’s Note

All claims expressed in this article are solely those of the authors and do not necessarily represent those of their affiliated organizations, or those of the publisher, the editors and the reviewers. Any product that may be evaluated in this article, or claim that may be made by its manufacturer, is not guaranteed or endorsed by the publisher.

## Supplementary Material

Supplementary Figure 1 Actin filament structure in conidia of WT, Δ*CgnoxA*, Δ*CgnoxB*, Δ*CgnoxR*, and Δ*CgnoxAnoxB* strains expressing Lifeact-EGFP. Scale bar = 5 μm.

Supplementary Figure 2 The relative expression level of Lifeact-GFP in the mutant strains. The β2-tubulin coding gene was used as the endogenous control for normalization and the relative expression level was estimated using the 2^-ΔΔCt^ method. Values are shown as the means ± standard deviations (SD).
