# Supplementary figures and images for: NADPH Oxidases Play a Role in Pathogenicity via the Regulation of F-Actin Organization in Colletotrichum gloeosporioides
Source: Front Cell Infect Microbiol. 2022 Jun 15;12:845133. doi: 10.3389/fcimb.2022.845133 (PMC9240266; doi:10.3389/fcimb.2022.845133)

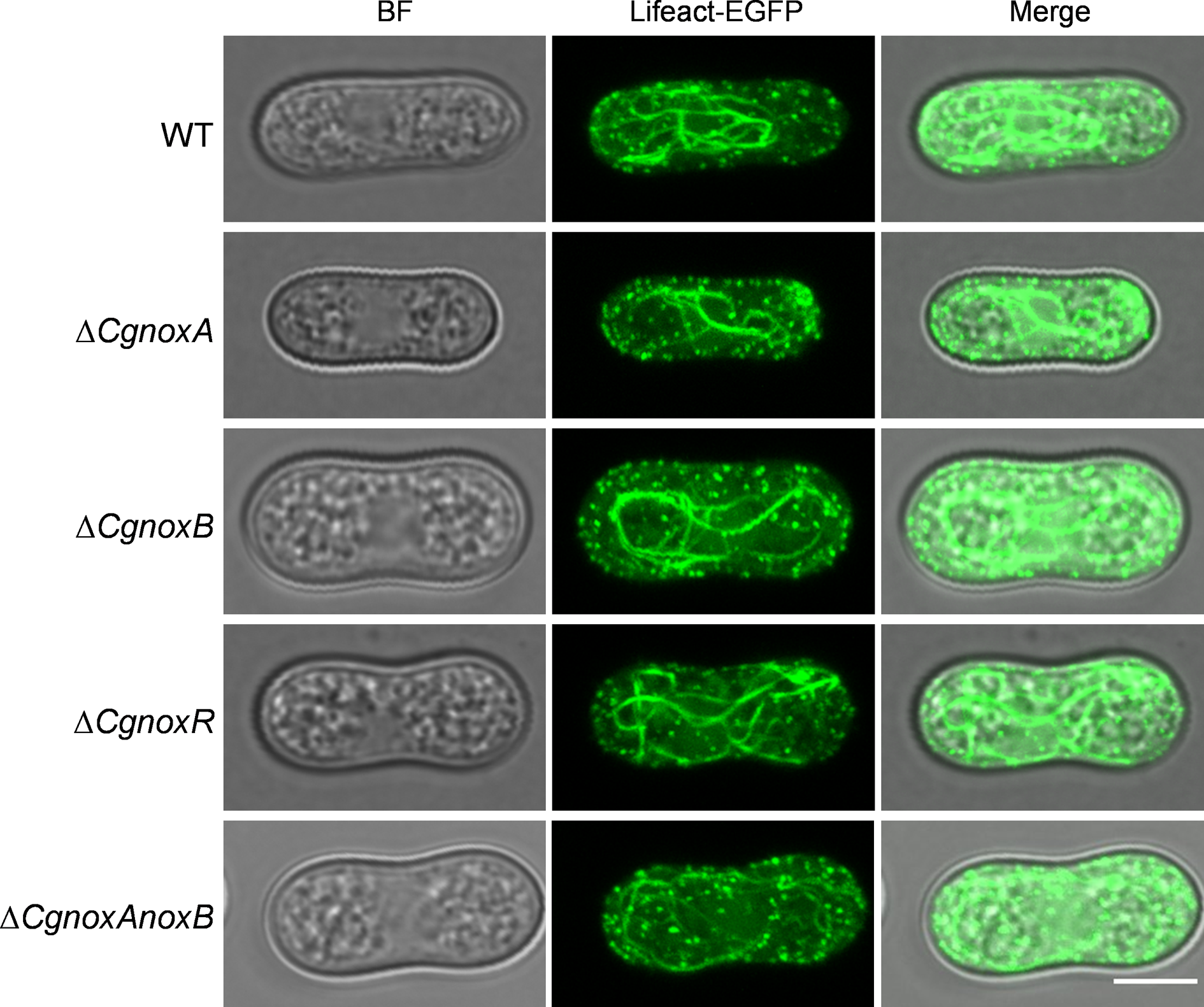

Supplement: Supplementary Figure 1 — Actin filament structure in conidia of WT, ΔCgnoxA, ΔCgnoxB, ΔCgnoxR, and ΔCgnoxAnoxB strains expressing Lifeact-EGFP. Scale bar = 5 μm. [file Image_1.tif]

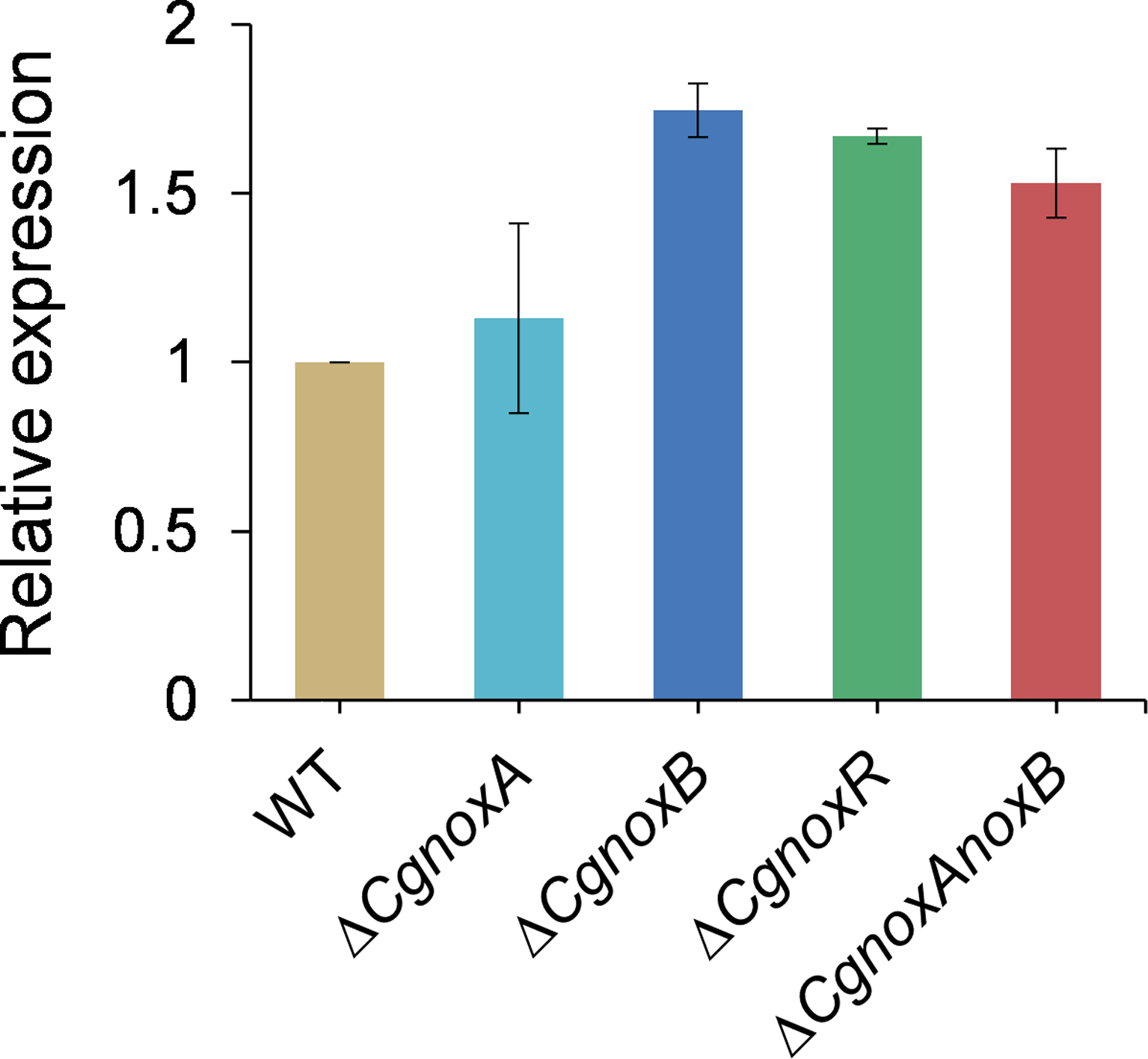

Supplement: Supplementary Figure 2 — The relative expression level of Lifeact-GFP in the mutant strains. The β2-tubulin coding gene was used as the endogenous control for normalization and the relative expression level was estimated using the 2-ΔΔCt method. Values are shown as the means ± standard deviations (SD). [file Image_2.tif]
